# Evaluating infection prevention and control programs in Zambian hospitals using the WHO infection prevention and control assessment framework tool

**DOI:** 10.3389/fpubh.2025.1642119

**Published:** 2025-08-29

**Authors:** Steward Mudenda, Joseph Yamweka Chizimu, Linus Ndegwa, Maisa Kasanga, Ilunga Mutwale, Aubrey Chichonyi Kalungia, Evelyn Wesangula, Adriano Focus Lubanga, James C. L. Mwansa, Martha Mwaba, Amos Yared Massele, Nyambe Sinyange, Tapfumanei Mashe, Malambo Mutila, Paul Simujayang’ombe, David Lowrance, Kaunda Yamba, Misheck Shawa, Chie Nakajima, Yasuhiko Suzuki, John Bwalya Muma, Massimo Sartelli, Brian Godman, Roma Chilengi

**Affiliations:** ^1^Antimicrobial Resistance Coordinating Committee, Zambia National Public Health Institute, Lusaka, Zambia; ^2^Department of Pharmacy, School of Health Sciences, University of Zambia, Lusaka, Zambia; ^3^Education and Continuous Professional Development Committee, Pharmaceutical Society of Zambia, Lusaka, Zambia; ^4^Infection Prevention Network-Kenya (IPNET-Kenya), Mombasa, Kenya; ^5^Department of Pathology and Microbiology, University Teaching Hospitals, Lusaka, Zambia; ^6^Department of Medicine, Kanyama Level 1 Hospital, Lusaka, Zambia; ^7^Strengthening Pandemic Preparedness, Eastern, Central, and Southern Africa Health Community, Arusha, Tanzania; ^8^Education and Research, Clinical Research Education and Management Services (CREAMS), Lilongwe, Malawi; ^9^Department of Clinical Services, Kamuzu Central Hospital (KCH), Lilongwe, Malawi; ^10^Department of Medicine, Lusaka Apex Medical University, Lusaka, Zambia; ^11^Resident Doctors Association of Zambia, Ministry of Health Zambia, Lusaka, Zambia; ^12^Sinazeze Hills Mini Hospital, Ministry of Health Zambia, Sinazongwe, Zambia; ^13^Department of Clinical Pharmacology and Therapeutics, Kairuki University, Dar Es Salaam, Tanzania; ^14^Health Systems Strengthening Unit, World Health Organization, Harare, Zimbabwe; ^15^The Global Fund, Geneva, Switzerland; ^16^Action on Antibiotic Resistance (ReAct) Africa, Lusaka, Zambia; ^17^Hokudai Center for Zoonosis Control in Zambia, Hokkaido University, Lusaka, Zambia; ^18^Division of Bioresources, Hokkaido University International Institute for Zoonosis Control, Sapporo, Japan; ^19^Department of Disease Control, School of Veterinary Medicine, University of Zambia, Lusaka, Zambia; ^20^Department of Surgery, Macerata Hospital, Macerata, Italy; ^21^Department of Public Health Pharmacy and Management, School of Pharmacy, Sefako Makgatho Health Sciences University, Ga-Rankuwa, South Africa; ^22^Department of Pharmacoepidemiology, Strathclyde Institute of Pharmacy and Biomedical Sciences, University of Strathclyde, Glasgow, United Kingdom; ^23^Antibiotic Policy Group, Institute for Infection and Immunity, City St. George’s, University of London, London, United Kingdom

**Keywords:** healthcare-associated infections, infection prevention and control, IPCAF, antimicrobial resistance, Zambia

## Abstract

**Background:**

Infection Prevention and Control (IPC) is key to preventing healthcare-associated infections (HAIs) and the spread of antimicrobial resistance (AMR). This study evaluated the implementation of IPC in Zambian hospitals.

**Materials and methods:**

We conducted a multicentric cross-sectional study in nine hospitals across Zambia using the WHO IPCAF tool. Data were collected from September 1 to 30, 2024 and analyzed using the self-scoring Excel and IBM SPSS version 25.0.

**Results:**

Out of the nine hospitals assessed, four were tertiary-level hospitals while the rest were secondary-level hospitals. Overall, the implementation of IPC core components was intermediate (IPCAF Score of 594 out of 800). Four hospitals had IPCAF scores between 401 and 600, indicating an intermediate level of IPC implementation. Five hospitals scored between 601 and 800, indicating an advanced implementation of IPC in these hospitals. Three tertiary hospitals scored between 601 and 800, demonstrating their advanced implementation of IPC core components.

**Conclusion:**

This study found that the overall implementation of IPC in the surveyed hospitals was intermediate, indicating that further improvements were needed. There is a need to provide peer-learning support and strengthen IPC implementation to respond to new or re-emerging infections and AMR in the country and beyond.

## Introduction

Infection prevention and control (IPC) is a systematic, data-driven practice aimed at curbing the transmission of preventable infections in the healthcare setting (1). It is a cornerstone of effective healthcare delivery, and essential for safeguarding patients and healthcare workers (2–4). In recent years, the growing threat of healthcare-associated infections (HAIs) and antimicrobial resistance (AMR) has underscored the urgent need for robust IPC programmes across health systems (5–9), particularly in low- and middle-income countries (LMICs) (10–14), including Zambia.

Healthcare-associated infections (HAIs) are a significant concern in the context of AMR, with virulent and high-risk microorganism strains such as “ESKAPE” pathogens – (*Enterococcus faecium, Staphylococcus aureus, Klebsiella pneumoniae, Acinetobacter baumannii, Pseudomonas aeruginosa and Enterobacter species*) of global significance (15–17). Evidence has indicated that HAIs are acquired by patients while receiving healthcare treatment (18, 19). These include Central Line-Associated Bloodstream Infections (CLABSI), Surgical Site Infections (SSI), respiratory infections like Hospital-Acquired Pneumonia (HAP), Ventilator-Associated Pneumonia (VAP), Catheter-Associated Urinary Tract Infections (CAUTI), gastrointestinal tract infections such as *Clostridioides difficile* infections (antibiotic-associated diarrhoea), and wound infections (18, 20–24). These infections are prevalent in intensive care units (ICUs), where patients are particularly vulnerable due to invasive procedures, immunocompromised, and extensive antibiotic use (5, 8, 15, 25).

The occurrence of these HAIs contributes to the overuse of antibiotics, increases costs and eventually the development and spread of AMR (15, 26). The economic and clinical burden associated with HAIs is substantial, with patients experiencing longer hospital stays and higher medical costs (27, 28). In the USA, attributable costs to HAIs have been estimated at $3,384 ($885–$7,717) per patient for vancomycin-resistant enterococci, increasing to $39,787 ($20,813–$64,140) for MDR *Acinetobacter* and further rising to $74,306 ($20,377–$128,235) for carbapenem-resistant (CR) *Acinetobacter* (29–31). Subsequently, AMR also increases patient morbidity and mortality and harms the global economy (26, 32–36).

The prevalence and burden of HAIs in LMICs are significant public health and economic concerns (5, 37, 38). Fraser et al. (39) estimated that the prevalence of HAIs across Africa between 2009 and 2018 ranged from 3 to 15% of patients; however, this was likely to be an underestimate with considerable under-reporting of HAIs across the continent (39). Abubakar et al. in 2022 estimated the prevalence of HAIs in Africa at 12.76%, with surgical site infections being the most common type (5). More recently, Hutton et al. (40) estimated that the number of HAIs among 14 sub-Saharan African countries was 4.8 million in 2022, with the number of deaths resulting from these estimated at 502,000. The total economic costs across the 14 sub-Saharan African countries were estimated to be at least US$13 billion in 2022, with the costs per capita of the population higher among lower-middle-income African countries at US$23.9 per capita versus low-income African countries at US$7.2 per capita (40).

Rates of HAIs are typically elevated across sub-Saharan Africa as a result of poor IPC practices due to limited resources, as well as typically overcrowding in hospitals, coupled with understaffing and underfunding of healthcare facilities (41, 42). Overall, it is estimated that up to 50% or more of public healthcare facilities across sub-Saharan Africa, especially in rural areas, lack routine access to clean water, basic hygiene, and basic waste management, adding to the development of HAIs (43–45). It is against these aforementioned challenges that strategies need to be instigated across countries to prevent infections and address AMR and its consequences (40, 46–56).

To help address the challenge of HAIs, the WHO developed the Infection Prevention and Control Assessment Framework (IPCAF) to evaluate and monitor IPC programs and support the implementation of IPC guidelines in healthcare facilities, particularly among LMICs (57–59). This user-friendly tool aligns with WHO’s eight core IPC components, providing a baseline assessment and enabling ongoing monitoring to track progress and identify gaps (57, 60). By guiding targeted interventions, the IPCAF aims to improve healthcare quality, reduce the level of HAIs, and combat AMR (58). Its structured approach supports data-driven IPC strategies, fostering accountability and continuous improvement, making it valuable, especially among LMICs where there are more concerns (4, 61–64).

Effective IPC is a multifaceted endeavor requiring a synergistic approach across various critical components (65, 66). Further, robust IPC programs, underpinned by evidence-based national and facility-level guidelines, standardize practices and ensure a systematic approach to safety (1, 57). Furthermore, comprehensive education and training empower healthcare workers (56), while continuous HAI surveillance (47, 67), coupled with diligent monitoring and feedback (57, 68), enables data-driven adjustments and fosters a culture of improvement. Alongside this, ensuring appropriate workload, staffing, and a well-maintained built environment with functional equipment provides the necessary infrastructure for safe care (59, 69–72). Collectively, these interconnected components are foundational to minimizing infection transmission, safeguarding patient safety, and playing a pivotal role in the global fight against AMR.

In Zambia, multidrug-resistant pathogens have been reported in hospitals, demonstrating the current significant burden of AMR (73–81). Very few studies, however, have been conducted on the extent of IPC implementation in Zambian hospitals (82, 83), hence this study. Furthermore, the implementation of IPC programmes in Zambia faces significant challenges, including resource limitations, inadequate infrastructure, and varying levels of IPC knowledge among healthcare workers. Notwithstanding, the authorities in Zambia have made notable strides toward improving IPC practices at the national and facility levels as part of ongoing initiatives to tackle AMR in the country as part of the National Action Plan (NAP) for AMR (84).

The adoption of tools like the IPCAF by the Antimicrobial Resistance Coordinating Committee (AMRCC) of the Zambia National Public Health Institute (ZNPHI) has the potential to further strengthen IPC programmes by providing a standardized mechanism to assess and improve practice. This is welcomed, given ongoing concerns regarding the excessive prescribing of antibiotics across different hospitals in Zambia (85–93). This study assessed the level and extent of IPC implementation in nine hospitals across Zambia. The findings of this study will inform the status of objective number three of the NAP and future directions toward preventing the occurrence of infections and AMR in Zambia (84).

## Materials and methods

### Study design, setting and population

**Figure 1:**
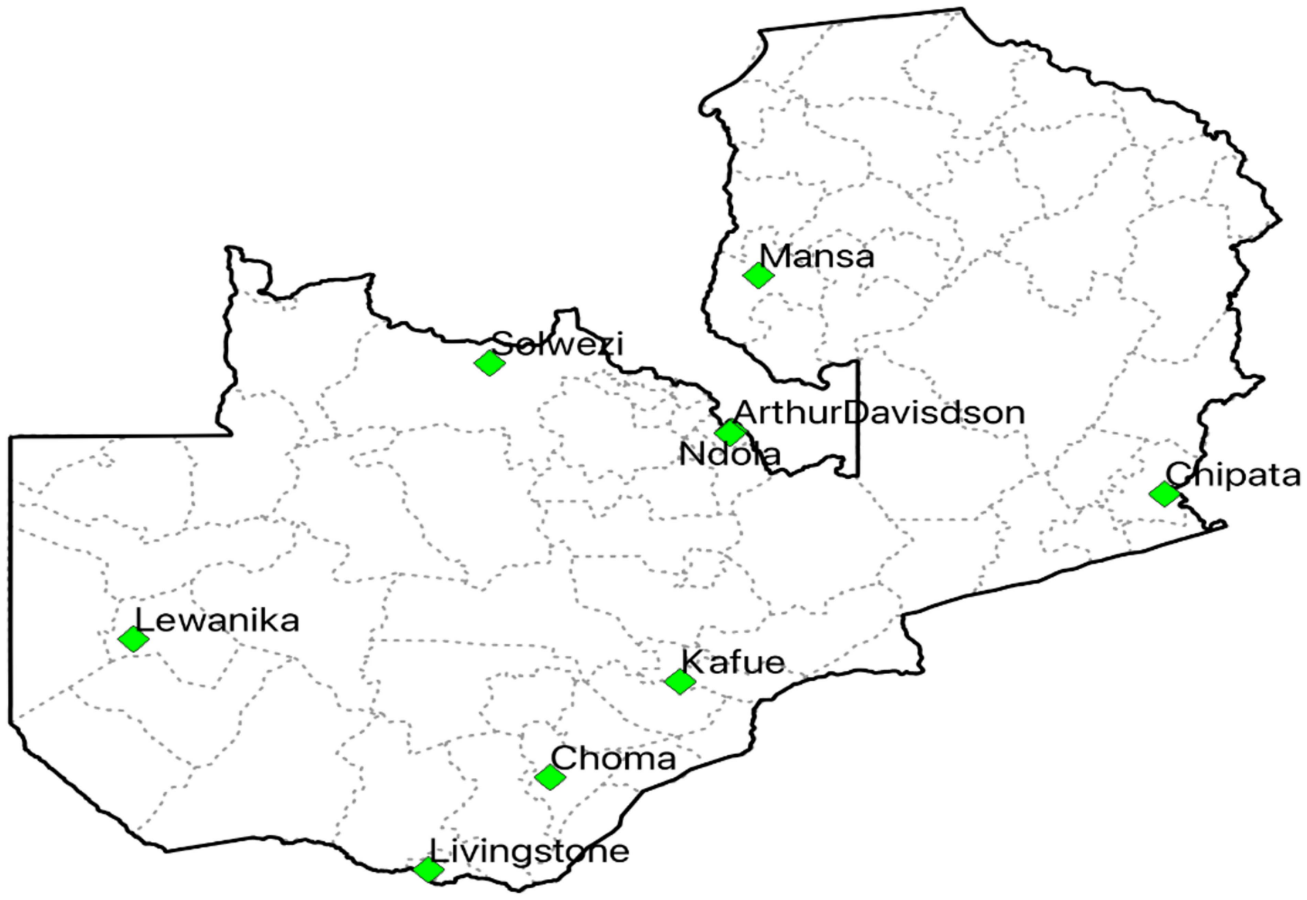
Map of Zambia indicating the IPC assessment study sites.

A self-reporting, descriptive multicentric cross-sectional survey was conducted to assess the IPC situation in hospitals in Zambia against the WHO minimum requirements for IPC programmes. The study was conducted from September 1 to 30, 2024 and included nine hospitals, out of which five were secondary-level public hospitals namely CGH, KGH, LGH, MGH, and SGH, respectively, and four were tertiary-level ADCH, CCH, LUTH, and NTH, respectively (Figure 1). In Zambia, Secondary-Level Hospitals, on the one hand, provide specialized referral services for areas of internal medicine, general surgery, paediatrics, obstetrics and gynecology, dentistry, psychiatry, and intensive care services. They are intended to serve a population range between 200,000 and 800,000, including referral patients from First-Level Hospitals. On the other hand, Tertiary-Level Hospitals provide medical services in internal medicine, surgery, paediatrics, obstetrics, gynecology, intensive care, and psychiatry, including health training and research. These hospitals are intended to serve a population of 800,000, including referrals from Secondary-Level Hospitals. The hospitals were selected using a purposive sampling method as they were implementing antimicrobial stewardship (AMS) programs to promote the rational use of antimicrobials, as reported in previous studies (94, 95). The nine hospitals included in this study were purposively selected to capture a range of facility types (secondary and tertiary) and geographical regions, with the aim of reflecting diverse IPC contexts within the country. However, these hospitals represent only a fraction of the total number of hospitals nationally, and therefore, the findings may not be fully representative of all healthcare facilities. To be eligible for inclusion, all hospitals were to be run and owned by the Government of the Republic of Zambia and with an established IPC Committee. Additionally, implementing effective IPC programmes in these hospitals is critical to prevent the occurrence and spread of infections and subsequently reduce the current overuse of antimicrobials. Furthermore, all targeted hospitals were secondary or tertiary-level. The targeted respondents were healthcare professionals or teams responsible for organizing and implementing IPC activities in the respective hospitals. Overall, 27 respondents were purposively selected from the respective IPC committees to respond to the questionnaire. This included the committee chairperson, secretary and IPC nurse from each of the nine hospitals.

### Data collection

Data collection was done using the pre-validated WHO IPCAF tool (57). We adopted the WHO globally validated IPCAF tool organized into eight IPC core components and multiple choice questions (57). The IPCAF is a globally validated evaluation tool designed to benchmark IPC performance at national and facility levels while supporting the implementation of the eight IPC core components (Supplementary material). The WHO IPCAF instrument used in this study was applied in its original form without local modifications. This decision was based on the instrument’s status as a globally standardized and validated tool designed for cross-country comparability. While the IPCAF has not undergone formal psychometric validation in Zambia, it has been widely used in LMIC settings with similar healthcare contexts, including in the African region. The research team ensured contextual relevance by providing brief clarifications during administration where necessary, without altering the structure, wording, or scoring system of the tool. Data collection was executed by 15 data collectors who were specifically trained for this purpose and the team visited each hospital for a period of 5 days translating into a 15-day data collection and dissemination period. Each data collection team visited three hospitals and conducted face-to-face interviews with the IPC committee members. The data collectors (medical doctors, pharmacists, nurses, public health staff, and biomedical scientists) were members of the IPC Technical Working Group (TWG) under the AMRCC who were trained on the use of the tool and had previous training on IPC using the national curriculum on AMS and IPC. The trainers were part of the Antimicrobial Resistance Coordinating Committee (AMRCC) who are experts in implementing IPC and AMS activities in Zambia. The training was done for a period of 3 days to ensure that the data collectors understood the need to collect complete and good quality data. REDCap accounts were opened for all the data collectors and testing of data entry was done on day two and three of training. After completion of the study, a meeting was held with the hospital management and staff where the findings were disseminated and recommendations were provided.

The IPCAF tool generates a final score ranging from 0 to 800, categorizing IPC implementation as inadequate, basic, intermediate, and advanced (57). This approach identifies gaps in current practices and fosters quality improvement initiatives. The scoring in the IPCAF tool is done as follows: Scores of 0–200 (Inadequate): IPC core component implementation is deficient and thus requires significant improvement; Scores of 201–400 (Basic): Some aspects of the IPC core components are in place, but not sufficiently implemented, hence, requires further improvement; Scores of 401–600 (Intermediate): Most aspects of IPC core components are appropriately implemented but there is a need to continue to improve the scope and quality of implementation and focus on the development of long-term plans to sustain and further promote the existing IPC program; Scores of 601–800 (Advanced): Means that the IPC core components are fully implemented according to the WHO recommendations and appropriate to the needs of the facility (57). All survey questions addressing the core components were multiple-choice, with response options such as “yes,” “no,” or “choose one answer.” The findings of the study were discussed with the IPC committee and disseminated across the surveyed hospitals.

### Data analysis

The collected data were checked for completeness before analysis. Data analysis was done using the self-scoring Excel sheet and IBM Statistical Package for Social Sciences (SPSS) version 25.0. Scoring for implementation of IPC core components was assigned as follows: 0–200 (Inadequate): 201–400 (Basic): 401–600 (Intermediate): and 601–800 (Advanced). Descriptive statistics were used to determine the overall implementation of IPC core components, and the status of IPC core components for each hospital was expressed as proportions. Additionally, descriptive statistics, including the mean and standard deviation, were used to summarize the overall IPCAF scores and the scores for each of the eight core components across the surveyed hospitals. Frequencies and percentages were used to describe the distribution of hospitals within the defined IPCAF implementation levels. To compare the mean IPC implementation scores across the nine hospitals, a One-Way Analysis of Variance (ANOVA) was performed. The statistical test (F-statistic) was conducted at a 95% confidence level and the level of statistical significance was set at *p* < 0.05.

### Ethical approval

The study was conducted in accordance with the Declaration of Helsinki, and approved by the Ethics Committee of Tropical Diseases Research Centre (TRC/C4/09/2023). Informed consent was obtained from all subjects involved in the study. Written informed consent has been obtained from the participants and facilities to publish this paper. Respondents and hospital management were informed that the findings of the study were to be published in peer-reviewed journals.

## Results

### Demographics of the surveyed hospitals

Overall, nine hospitals, including five (55.6%) secondary-level and four (44.4%) tertiary-level hospitals, were included in this study across seven provinces in Zambia, with an overall inpatient bed capacity of 3,300. Of the nine hospitals, two had bed capacities above 500, while the rest had between 150 and 420 beds (Table 1). The average bed capacity for secondary-level hospitals was 277 while that for tertiary-level hospitals was 479.

**Table 1:** Facility name, level of healthcare, and number of inpatient beds.

Hospital name	Province	Level	Number of beds
CGH	Southern	Secondary level	204
KGH	Lusaka	Secondary level	150
LGH	Western	Secondary level	310
MGH	Luapula	Secondary level	420
SGH	North-Western	Secondary level	300
ADCH	Copperbelt	Tertiary level	250
CCH	Eastern	Tertiary level	600
LUTH	Southern	Tertiary level	325
NTH	Copperbelt	Tertiary level	741

**Figure 2:**
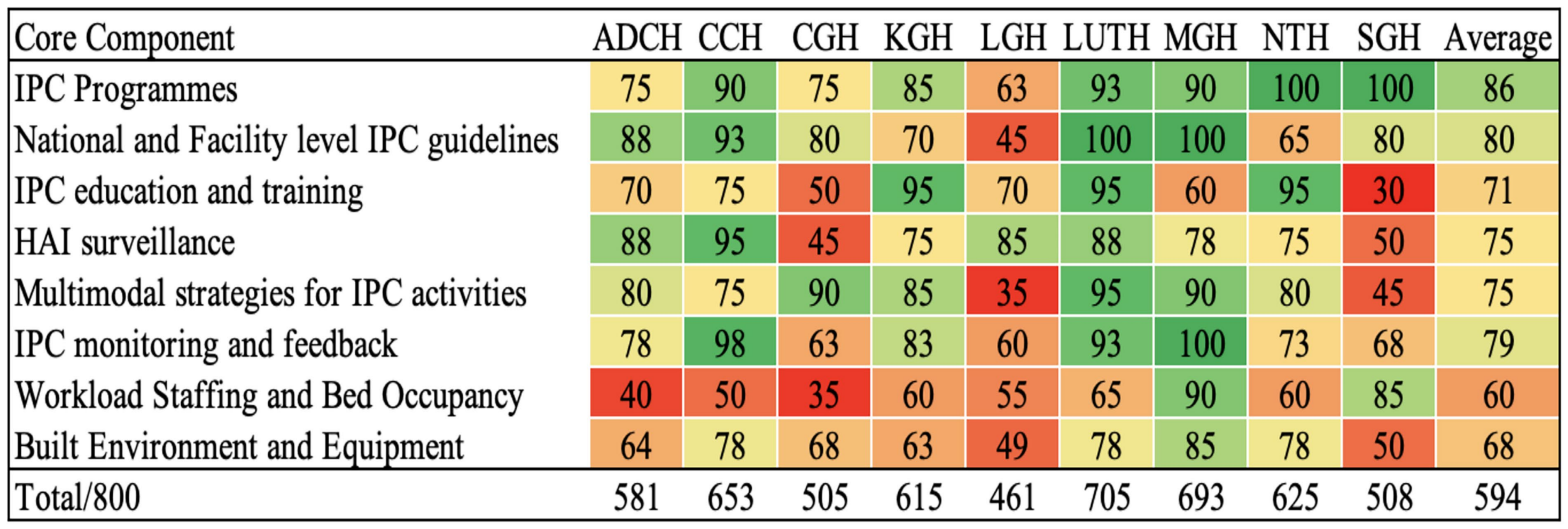
Distribution of IPCAF scores based on the surveyed hospitals.

The average overall IPCAF score across the hospitals was 594, indicating a generally intermediate level of IPC implementation (Figure 2). The distribution of hospital IPCAF scores revealed that 55.6% (*n =* 5) of the hospitals demonstrated advanced IPC implementation (score range 601–800), while 44.4% (*n =* 4) exhibited intermediate implementation (score range 401–600) (Figure 2). No hospitals scored within the basic level (<400). The F-statistic was found to be 3.72 and a *p* = 0.001, indicating that there is a statistically significant difference in the mean IPC implementation scores across the nine hospitals.

Tertiary hospitals outperformed secondary hospitals across most IPCAF core components, with the greatest differences observed in IPC education and training and HAI surveillance, reflecting stronger capacity-building initiatives and monitoring systems in higher-level facilities (Table 2). Secondary hospitals scored higher only in workload, staffing, and bed occupancy, likely due to lower patient volumes and reduced overcrowding. Both hospital categories demonstrated moderate performance in built environment, materials, and equipment for IPC, suggesting shared infrastructural gaps. The overall IPCAF score for tertiary hospitals was 641 compared to secondary hospitals at 556 (Table 2). The F-statistic was found to be 4.65 and a *p* = 0.049, indicating that there is a statistically significant difference in mean scores between secondary and tertiary hospitals, suggesting that hospital level is associated with variations in IPC performance across the IPCAF components.

**Table 2:** Scores for each of the eight core IPCAF components, stratified by hospital level.

IPCAF core component	Secondary hospitals (mean score)	Tertiary hospitals (mean score)
1. IPC Program	83	89
2. IPC Guidelines	75	86
3. IPC Education and Training	61	84
4. HAI Surveillance	67	86
5. Multimodal Strategies for IPC Implementation	69	83
6. Monitoring/Audit of IPC Practices and Feedback	75	85
7. Workload, Staffing, and Bed Occupancy	65	54
8. Built Environment, Materials, and Equipment for IPC at Facility Level	63	74
Overall IPCAF score by level of hospital	556	641

**Figure 3:**
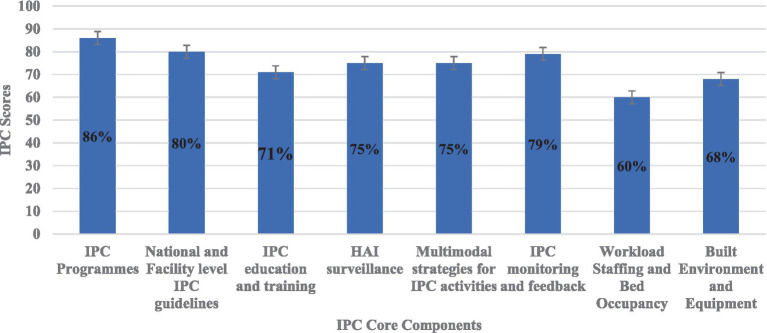
Percentage scores on implementation of IPC core component across the surveyed hospitals.

In this study, the IPC core components with the highest scores included IPC programmes (86%), National and Facility level IPC guidelines (80%), and IPC monitoring and feedback (79%) (Figure 3). Conversely, the IPC core components with the lowest scores included workload staffing and bed occupancy (60%), build environment and equipment (68%), IPC education and training (71%), HAI surveillance (75%), and multimodal strategies for IPC activities (75%) (Figure 3).

**Figure 4:**
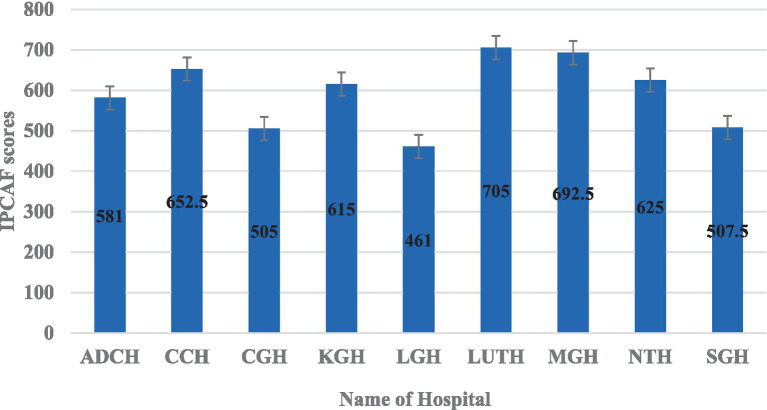
Overall IPCAF scores by hospital.

The hospital with the highest IPCAF score was LUTH (705) – a tertiary level hospital, followed by MGH (692.5), CCH (652.5), NTH (625), and KGH (615) all indicating an advanced level of IPC implementation (Figure 4). Consequently, the hospitals with the lowest IPCAF scores were LGH (461), CGH (505), SGH (507.5), and ADCH (581) which showed an intermediate implementation of IPC in these hospitals (Figure 4).

**Figure 5:**
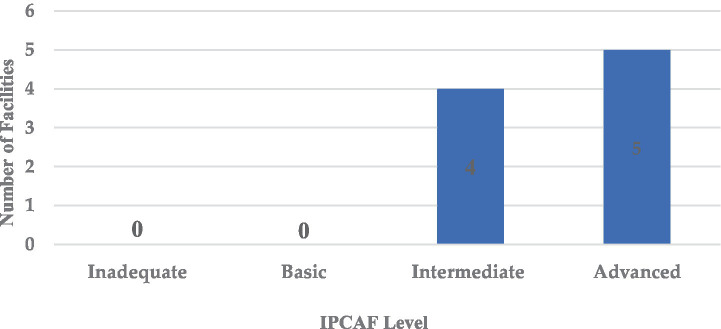
Summary of IPCAF levels of the nine hospitals included in the survey.

The IPCAF levels are shown in Figure 4 which shows that four hospitals (44.4%) had an intermediate IPCAF level while five hospitals (55.6%) had an advanced level (Figure 5).

Implementation of IPC in Zambia’s hospitals faces challenges such as limited dedicated staff, weak policy enforcement, insufficient funding, supply shortages, and inadequate training and infrastructure (Table 3). However, support from national guidelines, active committees, donor-funded programs, leadership commitment, and some infrastructure improvements act as facilitators to IPC efforts (Table 3).

**Table 3:** Systemic barriers and facilitators influencing IPC implementation in Zambia’s hospitals.

Domain	Barriers	Facilitators
Governance	Absence of dedicated IPC focal persons in some facilities Weak enforcement of national IPC policies and guidelines	Existing national IPC guidelines under the Ministry of Health Active IPC committees in some hospitals
Financing	Inadequate budget allocation for IPC activities Reliance on *ad hoc* or donor funding	Government and donor support for specific IPC programs
Supply Chain	Frequent shortages of PPE, disinfectants, and sterilization supplies Weak stock monitoring systems	Availability of some donor-supported procurement mechanisms
Human Resources	Limited IPC training opportunities High staff turnover impacting IPC continuity	Ongoing training initiatives supported by government and partners
Organizational Culture	Low prioritization of IPC by some hospital leadership Inconsistent staff adherence to IPC protocols	Strong leadership commitment in some facilities- Role modelling and advocacy by engaged leaders
Infrastructure	Inadequate isolation facilities and waste management systems	Infrastructure upgrades in certain hospitals through donor or government projects

## Discussion

To the best of our knowledge, this study represents the first nationwide assessment of the level and extent of IPC program implementation across different levels of public hospitals in the country. While previous reports (82, 83) may have touched upon aspects of IPC in specific settings, none have provided a comprehensive and validated nationwide assessment of IPC implementation. This study found an intermediate implementation of IPC core components in most secondary-level hospitals but an advanced implementation in most tertiary-level hospitals. Overall, the study found an intermediate implementation of IPC programs in the surveyed hospitals, which indicates a need to continue improving the scope and quality of IPC implementation. Alongside this, the focus should be on the development of long-term plans to sustain and further promote existing IPC programs in the country to reduce the prevalence of HAIs and the overuse of antibiotics among hospitals in Zambia.

The present study found an IPCAF score of 594, which indicates an intermediate level of IPC implementation in Zambian hospitals. These findings are similar to those reported across six hospitals in Uganda with an IPCAF score of 547 (96) and in Rwanda across 25 hospitals with an IPCAF score of 545 (97), potentially reflecting similar resource constraints and healthcare system structures within the East African region. Intermediate implementation of IPC, though lower than scores reported in Zambia, was also reported in Malawi across 33 hospitals with an IPCAF score of 445 (62) and in Burkina Faso with an IPCAF overall score of 415 (68), might indicate more significant challenges in basic IPC infrastructure and implementation in those contexts. Our findings are also better than those reported in the Democratic Republic of Congo (DRC) and Cote d’Ivoire. In the DRC, only a basic level implementation of IPC was reported, with an overall IPCAF score of 392.5 (68) while in Cote d’Ivoire, the score was only 242.5 (98), indicating the key barriers at play as reported in many LMICs, including inadequate allocation of budget for IPC, insufficient staffing of full-time IPC professionals, absence of clear IPC goals, challenges in staff training, no HAI surveillance, no periodic monitoring and inconsistent availability of IPC supplies (63, 99, 100). Conversely, the advanced implementation in Latin America (73) and high-income countries (HICs) (58, 60, 101–107) likely benefits from high-income level, greater investment in healthcare infrastructure, staffing, more attention given to IPC, and established IPC protocols (63). These findings indicate that most HICs have advanced implementation of IPC in their hospitals. Consequently, evidence has shown that significantly lower scores of IPC implementation were observed in LMICs and public healthcare facilities (58, 63).

Our study found that most tertiary-level hospitals had an advanced implementation of IPC core components compared to secondary-level hospitals. Arguably, this could be attributed to a higher density of specialized IPC-trained personnel serving in the facilities, more consistent access to funding for IPC supplies and programme support, or the presence of established IPC committees with more actions and implementing activities in the hospital. These findings underscores persistent disparities in resources and capacity that warrant targeted interventions to strengthen IPC performance nationwide. Our findings differ from those reported in Malawi, in which tertiary-level hospitals had an intermediate implementation of IPC programs (62). Our findings are also better than those reported in Bangladesh, where most tertiary-level hospitals had an inadequate implementation of IPC components (51). The findings in Malawi and Bangladesh, where tertiary hospitals showed intermediate implementation of IPC programs, suggest potentially different national-level resource allocation or policy implementation strategies.

Of the eight core components of IPC, the highest IPCAF scores were in IPC programmes and national and facility-level IPC guidelines. Our findings corroborate those reported in a study that was conducted in the DRC, in which the highest was the presence of IPC programmes, and national and facility IPC guidelines (68). This is in line with established global guidelines and the 17^th^ International Congress on Infectious Diseases workshop on establishing IPC and developing IPC resources for LMICs (108, 109). However, our findings differ from those reported in Burkina Faso, where the highest IPCAF scores were in HAI surveillance, built environment, and equipment (68). The difference could be due to the fact that in Zambia, IPC program guidelines scored well because they were instigated but the Zambian program had not implemented HAI surveillance. However, our findings guide future activities in this area in Zambia.

In our study, the lowest IPCAF scores were in workload, staffing, bed occupancy, built environment and equipment, and IPC education and training, which may reflect systemic issues within the Zambian healthcare system and limitations in infrastructure planning. Low scores in workload staffing and bed occupancy have also been reported in other LMICs, including the DRC and Malawi (62, 68). In Zambia, staffing, workload, and bed occupancy scored 60% better than the 45% reported in Malawi (62). Studies in Latin America, Germany, and Turkey also found low scores in workload, staffing and bed occupancy, followed by IPC training and multimodal strategies (102, 107, 110). Low scores in IPC education and training were also recorded in Burkina Faso (68). The low scores in IPC education and training highlight a crucial gap that has been recognized in other settings and likely hinders the effective implementation of IPC practices on the ground (111, 112). Providing education and training for healthcare workers in IPC is an essential area for the improvement of IPC practices and must be implemented in all countries (70). A study in China reported that multimodal strategies had the lowest average scores among the core components of IPC, thereby demonstrating gaps in adopting and using evidence-based strategies to implement IPC in hospitals (61).

The present study reported a 75% score in HAI surveillance. The scoring on HAI surveillance in our study may be biased due to the lack of participant understanding related to what constitutes HAI surveillance, because of the lack of training on HAI surveillance standards and requirements. This highlights a critical need for targeted education and capacity-building initiatives to ensure accurate data collection and effective utilization of HAI surveillance for IPC program improvement, as recommended by WHO guidelines on HAI surveillance (56, 109, 113). However, our findings are better than those reported in Malawi, where the HAI surveillance score was 40% (62). Our findings and those reported in other studies indicate the need to strengthen all the IPC core components in hospitals to control HAIs and prevent the emergence and spread of AMR. Furthermore, there is a need to develop IPC resources, especially for LMICs, where the burden of infections is high (108). Leadership support at the facility, national, and global levels is also needed to achieve implementation of the core components across all countries (109).

The gaps identified in IPC performance across the nine hospitals can be better understood by examining the underlying systemic barriers and facilitators. The study found that governance challenges, such as the absence of dedicated IPC focal persons in some facilities and weak enforcement of national guidelines, undermine consistent implementation of IPC policies. These gaps affect the full implementation of IPC strategies in hospitals, similar to findings from other studies (109, 114). For a functional and effective IPC programme, there is a need of having a dedicated focal point person to implement IPC measures (115). In the present study, inadequate financing, characterized by limited or non-ring-fenced budget allocations, restricts investment in essential supplies, infrastructure, and training. These challenges have been reported in other studies and affect the implementation of IPC in hospitals (99, 116–118). Our study further found that weaknesses in supply chain systems further exacerbate these constraints, with frequent shortages of PPE, disinfectants, and sterilization materials disrupting adherence to IPC protocols. Similar findings were reported in Nigeria where inadequate IPC materials affected the implementation of IPC in hospitals (119). The present study also found that in some hospitals, low prioritization of IPC by leadership and inconsistent staff compliance reflect competing demands, limited training opportunities, and insufficient role modelling. Lack of leadership support to implement IPC in hospitals has been reported to affect the effective prevention of infections (64, 120). Addressing these gaps is critical to full implementation of IPC strategies in hospitals.

Our study revealed several facilitators of IPC implementation in some hospitals, including the presence of active IPC committees, supportive leadership in certain facilities, and existing national guidelines that provide a structured framework for implementation. Donor-supported programs offering training and procurement support also helped to strengthen IPC in specific settings. These facilitators of IPC implementation have been recommended for LMICs, such as Zambia (59, 64). Therefore, these findings underscore the need for a multi-pronged approach that addresses governance, financing, supply chain resilience, and cultural change to close IPC performance gaps and sustain improvements over time.

We are aware that cross-sectional studies have limitations. Our study relied on self-reported measurements which may be prone to recall bias, including social desirability bias, whereby participants may overstate compliance with IPC standards, and recall bias, particularly for activities or events occurring in the past. The study was limited to nine hospitals, which may restrict the generalizability of the findings to other healthcare facilities across the country, particularly those with differing levels of resources, staffing, or patient populations. The purposive sampling approach may overrepresent facilities with better-established IPC programs or stronger administrative support, and underrepresent those with fewer resources. Alongside this, the cross-sectional nature of this study captured IPC performance at a single point in time within a month, which limits the ability to assess temporal changes, monitor trends, or establish causal relationships between identified factors and IPC outcomes. For example, infectious disease episodes may be higher during the rainy season, which may have an impact on the implementation of IPC strategies. These factors may have led to overestimation or underestimation of IPC performance in some hospitals. As a result, while the findings provide valuable insights into the current IPC landscape, they should be interpreted with caution and complemented by future longitudinal and observational studies. Notwithstanding, this study provides baseline findings that can be used to develop strategies for improving IPC in hospitals across Zambia. In doing so, this can impact reductions in antimicrobial use, and the emergence and spread of AMR. Future research should employ nationally representative longitudinal study designs that include both public and private hospitals across all provinces. Such studies would enhance the generalizability of findings, allow for the monitoring of IPC performance trends over time, and enable a more robust assessment of causal relationships between governance, financing, supply chain resilience, and IPC outcomes over time. Alongside this, future IPC research in Zambia should adopt mixed-methods approaches, combining quantitative assessments with qualitative studies to explore healthcare workers’ perspectives, organizational culture, and practical barriers to IPC adherence. Such approaches would provide deeper contextual understanding, uncover underlying systemic challenges, and inform the design of interventions that are both feasible and culturally appropriate.

### Policy recommendations and implications

The proposed policy recommendations address key systemic gaps identified in IPC implementation across Zambia’s hospitals (Table 4). Strengthening governance through the appointment of trained IPC focal persons and regular audit mechanisms would improve accountability and ensure adherence to national guidelines. Dedicated and ring-fenced funding at both national and facility levels is essential to sustain IPC programs and prioritize expenditure on critical supplies. Enhancing supply chain resilience via robust procurement systems and pooled purchasing arrangements would minimize shortages of essential IPC commodities. Building the skills and knowledge of healthcare workers through regular refresher training and integration of IPC into continuous professional development would reinforce best practices. Promoting a culture of safety through leadership advocacy, role modelling, and recognition systems would encourage staff adherence and institutional commitment. Finally, leveraging partnerships with development agencies for infrastructure upgrades, technical support, and collaborative research would expand the resource base and support innovation in IPC strategies. Together, these measures offer a practical and context-specific framework for improving IPC performance nationwide.

**Table 4:** Policy recommendations to strengthen infection prevention and control (IPC) in Zambia’s hospitals.

Policy area	Recommendation	Key actions
1. IPC governance	Strengthen governance and accountability for IPC	Appoint trained IPC focal persons in all hospitals Conduct regular audits and feedback sessions
2. Financing	Increase and ring-fence funding for IPC	Allocate dedicated budget lines at national and facility levels Prioritize spending on IPC essentials
3. Supply chain resilience	Ensure consistent availability of essential IPC commodities	Implement robust procurement and stock monitoring systems Explore pooled procurement with suppliers
4. Training and capacity building	Enhance skills and knowledge of healthcare workers	Institutionalize regular IPC refresher courses Integrate IPC into continuous professional development
5. Culture of safety	Promote a hospital-wide safety and IPC-oriented culture	Leadership advocacy and role modelling Recognition and reward systems for good IPC practices
6. Partnerships	Leverage partnerships for infrastructure and technical support	Collaborate with development partners for facility upgrades Support joint IPC research initiatives

## Conclusion

In conclusion, this study found an intermediate implementation of IPC core components in most secondary-level hospitals but an advanced implementation in most tertiary-level hospitals in Zambia. Overall, the average IPCAF level was found to be intermediate. The implementation of the WHO IPCAF tool in Zambia represents a significant opportunity to enhance IPC practices, reduce the burden of HAIs, and combat AMR. By leveraging this systematic and evidence-based approach, healthcare facilities can achieve measurable improvements in patient safety and healthcare quality. While this study provides valuable baseline data, future research should focus on conducting a larger, nationally representative survey, employing qualitative methods to explore barriers to IPC implementation at different hospital levels, and undertaking longitudinal studies to evaluate the impact of targeted IPC interventions on HAI rates and antibiotic use. Notably, our results contribute to ongoing efforts to optimize IPC programmes in Zambia, ensuring alignment with global best practices while addressing local healthcare challenges. Finally, there is a need for quality improvement in education and training of IPC measures among healthcare workers across all the hospitals in Zambia.

## Data Availability

The original contributions presented in the study are included in the article/Supplementary material, further inquiries can be directed to the corresponding author.
