# Supplementary material for: Evaluating infection prevention and control programs in Zambian hospitals using the WHO infection prevention and control assessment framework tool
Source: Front Public Health. 2025 Aug 29;13:1642119. doi: 10.3389/fpubh.2025.1642119 (PMC12425723; doi:10.3389/fpubh.2025.1642119)
Supplement: Supplementary file 1 [file Data_Sheet_1.pdf]

Infection Prevention and Control Assessment Framework (IPCAF) at the Facility Level

1. **Inadequate:** IPC core components implementation is deficient. Significant improvement is required.
2. **Basic:** Some aspects of the IPC core components are in place, but not sufficiently implemented. Further improvement is required.
3. **Intermediate:** Most aspects of the IPC core components are appropriately implemented. The facility should continue to improve the scope and quality of implementation and focus on the development of long-term plans to sustain and further promote the existing IPC programme activities.
4. **Advanced:** The IPC core components are fully implemented according to the WHO recommendations and appropriate to the needs of the facility.

Core component 1: Infection Prevention and Control (IPC) programme

Question	Answer	Score
**1. Do you have an IPC programme?**[3]<br>Choose one answer	☐ No	0
	☐ Yes, without clearly defined objectives	5
	☐ Yes, with clearly defined objectives and annual activity plan	10
**2. Is the IPC programme supported by an IPC team comprising of IPC professionals?**[4] Choose one answer	☐ No	0
	☐ Not a team, *only* an IPC focal person	5
	☐ Yes	10
**3. Does the IPC team have at least one full-time IPC professional or equivalent (nurse or doctor working 100% in IPC) available?**<br>Choose one answer	☐ No IPC professional available	0
	☐ No, *only* a part-time IPC professional available	2.5
	☐ Yes, one per > 250 beds	5
	☐ Yes, one per ≤ 250 beds	10
**4. Does the IPC team or focal person have dedicated time for IPC activities?**	☐ No	0
	☐ Yes	10
**5. Does the IPC team include both doctors and nurses?**	☐ No	0
	☐ Yes	10
**6. Do you have an IPC committee**[5] **actively supporting the IPC team?**	☐ No	0
	☐ Yes	10
**7. Are any of the following professional groups represented/included in the IPC committee?**		
Senior facility leadership (for example, administrative director, chief executive officer [CEO], medical director)	☐ No	0
	☐ Yes	5
Senior clinical staff (for example, physician, nurse)	☐ No	0
	☐ Yes	2.5
Facility management (for example, biosafety, waste, and those tasked with addressing water, sanitation, and hygiene [WASH])	☐ No	0
	☐ Yes	2.5
		0
		2.5
**8. Do you have clearly defined IPC objectives (that is, in specific critical areas)?**<br>Choose one answer	☐ No	
	☐ Yes, IPC objectives *only*	
	☐ Yes, IPC objectives and measurable outcome indicators (that is, adequate measures for improvement)	5
	☐ Yes, IPC objectives, measurable outcome indicators and set future targets	10
**9. Does the senior facility leadership show clear commitment and support for the IPC programme?**		
By an allocated budget specifically for the IPC programme (that is, covering IPC activities, including salaries)?	☐ No	0
	☐ Yes	5

By demonstrable support for IPC objectives and indicators within the facility (for example, at executive level meetings, executive rounds, participation in morbidity and mortality meetings)?	☐ No	0
	☐ Yes	5
**10. Does your facility have microbiological laboratory support (either present on or off site) for routine day-to-day use?** Choose one answer	☐ No	0
	☐ Yes, but not delivering results reliably (timely and of sufficient quality)	5
	☐ Yes, and delivering results reliably (timely and of sufficient quality)	10
**Subtotal score**		**/100**

3 IPC programmes should have clearly defined *objectives* based on local epidemiology and priorities according to risk assessment, and defined *functions and activities* that align with and contribute towards the prevention of health care-associated infections and antimicrobial resistance in health care. They should also include dedicated, trained IPC professionals. See the *WHO Guidelines on core components of IPC programmes at the national and acute health care facility level* for more information (**http://www.who.int/infection-prevention/publications/core-components/en/**, accessed 13 April 2018).

4 IPC professional: medical or nursing staff trained in a certified IPC course.

5 An IPC committee is a multidisciplinary group with interested stakeholders across the facility, which interacts with and advises the IPC team. An IPC team includes dedicated IPC professionals who are responsible for the IPC programme.

Core component 2: Infection Prevention and Control (IPC) guidelines

Question	Answer	Score
**1. Does your facility have the expertise (in IPC and/or infectious diseases) for developing or adapting guidelines?**	☐ No	0
	☐ Yes	7.5
**2. Does your facility have guidelines available for:**		
Standard precautions?	☐ No	0
	☐ Yes	2.5
Hand hygiene?	☐ No	0
	☐ Yes	2.5
Transmission-based precautions?[6]	☐ No	0
	☐ Yes	2.5
Outbreak management and preparedness?	☐ No	0
	☐ Yes	2.5
Prevention of surgical site infection?[7]	☐ No	0
	☐ Yes	2.5
Prevention of vascular catheter-associated bloodstream infections?	☐ No	0
	☐ Yes	2.5
Prevention of hospital-acquired pneumonia ([HAP]; all types of HAP, including (but not exclusively) ventilator-associated pneumonia)?	☐ No	0
	☐ Yes	2.5
Prevention of catheter-associated urinary tract infections?	☐ No	0
	☐ Yes	2.5
Prevention of transmission of multidrug-resistant (MDR) pathogens?	☐ No	0
	☐ Yes	2.5
Disinfection and sterilization?	☐ No	0
	☐ Yes	2.5
Health care worker protection and safety[8]	☐ No	0
	☐ Yes	2.5
Injection safety?	☐ No	0

	☐ Yes	2.5
Waste management?	☐ No	0
	☐ Yes	2.5
Antibiotic stewardship?[9]	☐ No	0
	☐ Yes	2.5

6 Transmission-based Precautions are to be used in addition to Standard Precautions for patients who may be infected or colonized with certain infectious agents for which additional precautions are needed to prevent infection transmission. They are based on the routes of transmission of specific pathogens (for example, contact versus droplets). More information can be found in the United States Centers for Disease Control and Prevention Guidelines for Isolation Precautions (**https://www.cdc.gov/infectioncontrol/pdf/guidelines/isolation-guidelines.pdf**, accessed 13 April 2018).

7 If no surgical interventions are undertaken at your facility, choose answer "Yes".

8 Includes aspects of improving working conditions, detection of occupational diseases, health surveillance of workers, pre-employment screening and vaccinations.

9 Refers to the appropriate use of antimicrobials to improve patient outcomes while minimizing the development and spread of resistance. More information can be found in the *WHO Global Framework for Development & Stewardship to Combat Antimicrobial Resistance* (**http://www.who.int/phi/implementation/research/UpdatedRoadmap-Global-Framework-for-Development-Stewardship-tocombatAMR_2017_11_03.pdf?ua=1**, accessed 29 March 2018).

**3. Are the guidelines in your facility consistent with national/international guidelines (if they exist)?**	☐ No	0
	☐ Yes	10
**4. Is implementation of the guidelines adapted[10] according to the local needs and resources while maintaining key IPC standards?**	☐ No	0
	☐ Yes	10
**5. Are frontline health care workers involved in <u>both</u> planning and executing the implementation of IPC guidelines in addition to IPC personnel?**	☐ No	0
	☐ Yes	10
**6. Are relevant stakeholders (for example, lead doctors and nurses, hospital managers, quality management) involved in the development and adaptation of the IPC guidelines in addition to IPC personnel?**	☐ No	0
	☐ Yes	7.5
**7. Do health care workers receive specific training related to new or updated IPC guidelines introduced in the facility?**	☐ No	0
	☐ Yes	10
**8. Do you regularly monitor the implementation of at least some of the IPC guidelines in your facility?**	☐ No	0
	☐ Yes	10
**Subtotal score**		**/100**

10 IPC team carefully reviews guidelines to prioritize activities according to needs and resources while maintaining key IPC standards.

Core component 3: Infection Prevention and Control (IPC) education and training

Question	Answer	Score
**1. Are there personnel with the IPC expertise (in IPC and/or infectious diseases) to lead IPC training?**	☐ No	0
	☐ Yes	10
**2. Are there additional non-IPC personnel with adequate skills to serve as trainers and mentors (for example, link nurses or doctors, champions)?** Choose one answer	☐ No	0
	☐ Yes	10
**3. How frequently do health care workers receive training regarding IPC in your facility?** Choose one answer	☐ Never or rarely	0
	☐ New employee orientation *only* for health care workers	5
	☐ New employee orientation and regular (at least annually) IPC training for health care workers offered but not mandatory	10
	☐ New employee orientation and regular (at least annually) mandatory IPC training for all health care workers	15
**4. How frequently do cleaners and other personnel directly involved in patient care receive training regarding IPC in your facility?** Choose one answer	☐ Never or rarely	0
	☐ New employee orientation *only* for other personnel	5
	☐ New employee orientation and regular (at least annually) training for other personnel offered but not mandatory	10
	☐ New employee orientation and regular (at least annually) mandatory IPC training for other personnel	15
**5. Does administrative and managerial staff receive general training regarding IPC in your facility?** Choose one answer	☐ No	0
	☐ Yes	5
**6. How are health care workers and other personnel trained?** Choose one answer	☐ No training available	0
	☐ Using written information and/or oral instruction and/or e-learning *only*	5
	☐ Includes *additional* interactive training sessions (for example, simulation and/or bedside training)	10
**7. Are there periodic evaluations of the effectiveness of training programmes (for example, hand hygiene audits, other checks on knowledge)?** Choose one answer	☐ No	0
	☐ Yes, but not regularly	5
	☐ Yes, regularly (at least annually)	10
**8. Is IPC training integrated in the clinical practice and training of other specialties (for example, training of surgeons involves aspects of IPC)?** Choose one answer	☐ No	0
	☐ Yes, in some disciplines	5
	☐ Yes, in all disciplines	10
**9. Is there specific IPC training for patients or family members to minimize the potential for health care-associated infections (for example, immunosuppressed patients, patients with invasive devices, patients with multidrug-resistant infections)?**	☐ No	0
	☐ Yes	5
**10. Is ongoing development/education offered for IPC staff (For example, by regularly attending conferences, courses)?**	☐ No	0
	☐ Yes	10
**Subtotal score**		**/100**

Core component 4: Health care-associated infection (HAI) surveillance

Question	Answer	Score
**Organization of surveillance**		

**1. Is surveillance a defined component of your IPC programme?**	☐ No	0
	☐ Yes	5
**2. Do you have personnel responsible for surveillance activities?**	☐ No	0
	☐ Yes	5
**3. Have the professionals responsible for surveillance activities been trained in basic epidemiology, surveillance and IPC (that is, capacity to oversee surveillance methods, data management and interpretation)?**	☐ No	0
	☐ Yes	5
**4. Do you have informatics/IT support to conduct your surveillance (for example, equipment, mobile technologies, electronic health records)?**	☐ No	0
	☐ Yes	5
**Priorities for surveillance - defined according to the scope of care**		
**5. Do you go through a prioritization exercise to determine the HAIs to be targeted for surveillance according to the local context (that is, identifying infections that are major causes of morbidity and mortality in the facility)?**[11]	☐ No	0
	☐ Yes	5
**6. In your facility is surveillance conducted for:**		
Surgical site infections?[12]	☐ No	0
	☐ Yes	2.5
Device-associated infections (for example, catheter-associated urinary tract infections, central line-associated bloodstream infections, peripheral-line associated bloodstream infections, ventilator-associated pneumonia)?	☐ No	0
	☐ Yes	2.5
Clinically-defined infections (for example, definitions based only on clinical signs or symptoms in the absence of microbiological testing)?	☐ No	0
	☐ Yes	2.5
Colonization or infections caused by multidrug-resistant[13] pathogens according to your local epidemiological situation?	☐ No	0
	☐ Yes	2.5
Local priority epidemic-prone infections (for example, norovirus, influenza, tuberculosis [TB], severe acute respiratory syndrome [SARS], Ebola, Lassa fever)?	☐ No	0
	☐ Yes	2.5
Infections in vulnerable populations (for example, neonates, intensive care unit, immunocompromised, burn patients)?[14]	☐ No	0
	☐ Yes	2.5
Infections that may affect health care workers in clinical, laboratory, or other settings (for example, hepatitis B or C, human immunodeficiency virus [HIV], influenza)?	☐ No	0
	☐ Yes	2.5
**7. Do you regularly evaluate if your surveillance is in line with the current needs and priorities of your facility?**[11]	☐ No	0
	☐ Yes	5

11 A prioritization exercise should be undertaken to determine which HAIs to target for surveillance according to the local context (for example, areas and/or patients most at risk) according to available resources (see *Interim practical manual* supporting implementation of the WHO Guidelines on Core Components of Infection Prevention and Control Programmes at http://www.who.int/infectionprevention/tools/core-components/en/, accessed 3 May 2018)

12 If no surgical interventions are undertaken at your facility, choose answer "Yes".

13 Multidrug-resistant: Non-susceptibility to at least one agent in three or more antimicrobial categories;14 If vulnerable patient populations are not treated at your facility, choose answer "Yes".

**Methods of surveillance**		
**8. Do you use reliable surveillance case definitions (defined numerator and denominator according to international definitions [e.g. CDC NHSN/ECDC]**[15] **or if adapted, through an evidence-based adaptation process and expert consultation?**	☐ No	0
	☐ Yes	5

**9. Do you use standardized data collection methods (for example, active prospective surveillance) according to international surveillance protocols (for example, CDC NHSN/ECDC) or if adapted, through an evidence-based adaptation process and expert consultation?**	☐ No	0
	☐ Yes	5
**10. Do you have processes in place to regularly review data quality (for example, assessment of case report forms, review of microbiology results, denominator determination, etc.)?**	☐ No	0
	☐ Yes	5
**11. Do you have adequate microbiology and laboratory capacity to support surveillance?** Choose one answer	☐ No	0
	☐ Yes, can differentiate gram-positive/negative strains but cannot identify pathogens	2.5
	☐ Yes, can reliably identify pathogens (for example, isolate identification) in a timely manner	5
	☐ Yes, can reliably identify pathogens and antimicrobial drug resistance patterns (that is, susceptibilities) in a timely manner	10
**Information analysis and dissemination/data use, linkage, and governance**		
**12. Are surveillance data used to make tailored unit/facility-based plans for the improvement of IPC practices?**	☐ No	0
	☐ Yes	5
**13. Do you analyze antimicrobial drug resistance on a regular basis (for example, quarterly/half-yearly/annually)?**	☐ No	0
	☐ Yes	5
**14. Do you regularly (for example, quarterly/half-yearly/annually) feedback up-to-date surveillance information to:**		
Frontline health care workers (doctors/nurses)?	☐ No	0
	☐ Yes	2.5
Clinical leaders/heads of department	☐ No	0
	☐ Yes	2.5
IPC committee	☐ No	0
	☐ Yes	2.5
Non-clinical management/administration (chief executive officer/chief financial officer)?	☐ No	0
	☐ Yes	2.5
**15. How do you feedback up-to-date surveillance information? (at least annually)** Choose one answer	☐ No feedback	0
	☐ By written/oral information *only*	2.5
	☐ By presentation and interactive problem-orientated solution finding	7.5
**Subtotal score**		**/100**

15 United States Centers for Disease Control and Prevention (CDC) National Healthcare Safety Network (NHSN) (**https://www.cdc.gov/nhsn/index.html**, accessed 13 April 2018); European Centre for Disease Prevention and Control (ECDC) (**https://ecdc.europa.eu/en/about-us/partnerships-and-networks/disease-and-laboratory-networks/hai-net**, accessed 13 April 2018).

Core component 5: Multimodal strategies[16] for implementation of infection prevention and control (IPC) interventions

Question	Answer	Score
	☐ No	0

**1. Do you use multimodal strategies[16] to implement IPC interventions?**	☐ Yes	15
**2. Do your multimodal strategies include any or all of the following elements:** Choose one answer (the most accurate) per element	**System change**	0
	☐ Element not included in multimodal strategies	0
	☐ Interventions to ensure the necessary infrastructure and continuous availability of supplies are in place	5
	☐ Interventions to ensure the necessary infrastructure and continuous availability of supplies are in place and addressing ergonomics[17] and accessibility, such as the best placement of central venous catheter set and tray	10
	**Education and training**	
	☐ Element not included in multimodal strategies	0
	☐ Written information and/or oral instruction and/or e-learning *only*	5
	☐ *Additional* interactive training sessions (includes simulation and/or bedside training)	10
	**Monitoring and feedback**	
	☐ Element not included in multimodal strategies	0
	☐ Monitoring compliance with process or outcome indicators (for example, audits of hand hygiene or catheter practices)	5
	☐ Monitoring compliance and providing timely feedback of monitoring results to health care workers and key players	10
	**Communications and reminders**	
	☐ Element not included in multimodal strategies	0
	☐ Reminders, posters, or other advocacy/awareness-raising tools to promote the intervention	5
	☐ *Additional* methods/initiatives to improve team communication across units and disciplines (for example, by establishing regular case conferences and feedback rounds)	10
	**Safety climate and culture change**	
	☐ Element not included in multimodal strategies	0
	☐ Managers/leaders show visible support and act as champions and role models, promoting an adaptive approach[18] and strengthening a culture that supports IPC, patient safety and quality	5
	☐ *Additionally*, teams and individuals are empowered so that they perceive ownership of the intervention (for example, by participatory feedback rounds)	10

16 See definition at **http://www.who.int/infection-prevention/publications/ipc-cc-mis.pdf?ua=1**, accessed 13 April 2018. The use of multimodal strategies in IPC has been shown to be the best evidencebased approach to achieve sustained system and behavioural change for the implementation of IPC interventions. Multimodal strategy: ≥3 components implemented in an integrated way to achieve improvement of an outcome and change behavior (for example, hand hygiene practices). Components can include (i) system change (for example, making the necessary infrastructure, supplies and human resources available), (ii) education and training of health care workers and key players (for example, managers), (iii) monitoring infrastructures, practices, processes, outcomes and providing data feedback; (iv) reminders in the workplace/communications; and (v) culture change within the establishment or the strengthening of a safety climate. It also includes tools, such as checklists and bundles, developed by multidisciplinary teams that take into account local conditions. All five areas should be considered and necessary action taken, based on the local context and situation informed by periodic assessments. Lessons from the field of implementation science suggest that targeting only one of these five elements (that is, using a "unimodal" strategy) is more likely to result in improvements that are short-lived and not sustainable.
For more information, please see: **http://www.who.int/infection-prevention/publications/ipc-cc-mis.pdf?ua=1**, accessed 13 April 2018 and the Interim practical manual supporting implementation of the WHO Guidelines on Core Components of Infection Prevention and Control Programmes at **http://www.who.int/infection-prevention/tools/core-components/en/**, accessed 3 April 2018.

17 Ergonomics: human factors or an understanding of interactions among humans and elements of a system to optimize human well-being and overall system performance and prevent human error. More information at: http://www.health.org.uk/sites/health/files/IntegratingHumanFactorsWithInfectionAndPreventionControl.pdf, accessed 13 April 2018.

18 Adaptive approaches consider the behavioural, organizational and cultural complexity in health care systems. They aim to improve the local safety climate and motivate local teams to consistently perform best practices by shaping attitudes, beliefs, and values of clinicians. This could include engaging leadership, improving collaborations and team work, and facilitating staff ownership of the intervention. More information at: **https://www.ahrq.gov/professionals/education/curriculum-tools/cusptoolkit/index.html**, accessed 13 April 2018.

**3. Is a multidisciplinary team used to implement IPC multimodal strategies?**	☐ No	0
	☐ Yes	15
**4. Do you regularly link to colleagues from quality improvement and patient safety to develop and promote IPC multimodal strategies?**	☐ No	0
	☐ Yes	10
**5. Do these strategies include bundles[19] or checklists?**	☐ No	0

	☐ Yes	10
**Subtotal score**		**/100**

19 Bundles: sets of evidence-based practices focused on improving the care process in a structured manner, for example, improvement of catheter insertion. Please note that bundles and multimodal strategies are not the same concept; bundles are tools that can be used to facilitate the implementation of IPC measures, ideally in the context of multimodal strategies[16] which are a much more comprehensive approach.

Core component 6: Monitoring/audit of IPC practices and feedback

Question	Answer	Score
**1. Do you have trained personnel responsible for monitoring/audit of IPC practices and feedback?**	☐ No	0
	☐ Yes	10
**2. Do you have a well-defined monitoring plan with clear goals, targets and activities (including tools to collect data in a systematic way)?**	☐ No	0
	☐ Yes	7.5
**3. Which processes and indicators do you monitor in your facility?** Tick all that apply	☐ None	0
	☐ Hand hygiene compliance (using the WHO hand hygiene observation tool[20] or equivalent)	5
	☐ Intravascular catheter insertion and/or care	5
	☐ Wound dressing change	5
	☐ Transmission-based precautions and isolation to prevent the spread of multidrug resistant organisms (MDRO)	5
	☐ Cleaning of the ward environment	5
	☐ Disinfection and sterilization of medical equipment/instruments	5
	☐ Consumption/usage of alcohol-based handrub or soap	5
	☐ Consumption/usage of antimicrobial agents	5
	☐ Waste management	5
**4. How frequently is the *WHO Hand Hygiene Self-Assessment Framework Survey*[21] undertaken?** Choose one answer	☐ Never	0
	☐ Periodically, but no regular schedule	2.5
	☐ At least annually	5
**5. Do you feedback auditing reports (for example, feedback on hand hygiene compliance data or other processes) on the state of the IPC activities/performance?** Tick all that apply	☐ No reporting	0
	☐ Yes, within the IPC team	2.5
	☐ Yes, to department leaders and managers in the areas being audited	2.5
	☐ Yes, to frontline health care workers	2.5
	☐ Yes, to the IPC committee or quality of care committees or equivalent	2.5
	☐ Yes, to hospital management and senior administration	2.5
**6. Is the reporting of monitoring data undertaken regularly (at least annually)?**	☐ No	0
	☐ Yes	10
**7. Are monitoring and feedback of IPC processes and indicators performed in a "blame-free" institutional culture aimed at improvement and behavioural change?**	☐ No	0
	☐ Yes	5
**8. Do you assess safety cultural factors in your facility (for example, by using other surveys such as HSOPSC, SAQ, PSCHO, HSC[22])**	☐ No	0
	☐ Yes	5
**Subtotal score**		**/100**

20 WHO hand hygiene monitoring and feedback tools can be found here: http://www.who.int/infection-prevention/tools/hand-hygiene/evaluation_feedback/en/, accessed 18 April 2018.

21 *WHO Hand Hygiene Self-Assessment Framework* can be found here: http://www.who.int/gpsc/country_work/hhsa_framework_October_2010.pdf?ua=1, accessed 18 April 2018.

22 HSOPSC: Hospital survey on patient safety culture; SAQ: Safety attitudes questionnaire, PSCHO: Patient safety climate in healthcare organizations; HSC: Hospital safety climate scale. A summary of these surveys can be found at: Colla JB, et al. Measuring patient safety climate: a review of survey. Qual Saf Health Care. 2005;14(5):364-6 (https://www.ncbi.nlm.nih.gov/pubmed/16195571, accessed 13 April 2018).

Core component 7: Workload, staffing and bed occupancy[23]

Question	Answer	Score
**Staffing**		
**1. Are appropriate staffing levels assessed in your facility according to patient workload using national standards or a standard staffing needs assessment tool such as the *WHO Workload indicators of staffing need*[24] method?**	☐ No	0
	☐ Yes	5
**2. Is an agreed (that is, WHO or national) ratio of health care workers to patients[25] maintained across your facility?** Choose one answer	☐ No	0
	☐ Yes, for staff in less than 50% of units	5
	☐ Yes, for staff in more than 50% of units	10
	☐ Yes, for all health care workers in the facility	15
**3. Is a system in place in your facility to act on the results of the staffing needs assessments when staffing levels are deemed to be too low?**	☐ No	0
	☐ Yes	10
**Bed occupancy**		
**4. Is the design of wards in your facility in accordance with international standards[26] regarding bed capacity?** Choose one answer	☐ No	0
	☐ Yes, <u>but</u> *only* in certain departments	5
	☐ Yes, for all departments (including emergency department and pediatrics)	15
**5. Is bed occupancy in your facility kept to one patient per bed?** Choose one answer	☐ No	0
	☐ Yes, <u>but</u> *only* in certain departments	5
	☐ Yes, for all units (including emergency departments and pediatrics)	15
**6. Are patients in your facility placed in beds standing in the corridor outside of the room (including beds in the emergency department)?** Choose one answer	☐ Yes, more frequently than twice a week	0
	☐ Yes, less frequently than twice a week	5
	☐ No	15
**7. Is adequate spacing of > 1 meter between patient beds ensured in your facility?** Choose one answer	☐ No	0
	☐ Yes, <u>but</u> *only* in certain departments	5
	☐ Yes, for all departments (including emergency department and pediatrics)	15
**8. Is a system in place in your facility to assess and respond when adequate bed capacity is exceeded?** Choose one answer	☐ No	0
	☐ Yes, this is the responsibility of the head of department	5
	☐ Yes, this is the responsibility of the hospital administration/ management	10
**Subtotal score**		**/100**

23 Particularly for these questions, the IPC team may need to consult with other relevant teams in the facility to be able to respond to questions accordingly.

24 The *WHO Workload indicators of staffing need* method provides health managers with a systematic way to determine how many health workers of a particular type are required to cope with the workload of a given health facility and aid decision-making (**http://www.who.int/hrh/resources/wisn_user_manual/en/**, accessed 13 April 2018).

25 Taking into account all health care workers involved in service delivery and patient care, including clinical staff (doctors, nurses, dentists, medical assistants, etc.), laboratory technicians and other health care workers (for example, cleaners).

26 The *WHO Essential environmental health standards in health care guidance* provides guidance on standards required for health care in medium- and low-resource countries. These guidelines have been written for use by health managers and planners, architects, urban planners, water and sanitation staff, clinical and nursing staff, carers and other health care providers, and health promoters (**http://www.who.int/water_sanitation_health/publications/ehs_hc/en/**, accessed 13 April 2018).

Core component 8: Built environment, materials and equipment for IPC at the facility level[27]

Question	Answer	Score
**Water**		
	☐ No, available on average < 5 days per week	0

**1. Are water services available at all times and of sufficient quantity for all uses (for example, hand washing, drinking, personal hygiene, medical activities, sterilization, decontamination, cleaning and laundry)?** Choose one answer	☐ Yes, available on average ≥ 5 days per week or every day but not of sufficient quantity	2.5
	☐ Yes, every day and of sufficient quantity	7.5
**2. Is a reliable safe drinking water station present and accessible for staff, patients and families at all times and in all locations/wards?** Choose one answer	☐ No, not available	0
	☐ Sometimes, or only in some places or not available for all users	2.5
	☐ Yes, accessible at all times and for all wards/groups	7.5
**Hand hygiene and sanitation facilities**		
**3. Are functioning hand hygiene stations (that is, alcohol-based handrub solution or soap and water and clean single-use towels) available at all points of care?** Choose one answer	☐ No, not present	0
	☐ Yes, stations present, but supplies are not reliably available	2.5
	☐ Yes, with reliably available supplies	7.5
**4. In your facility, are ≥ 4 toilets or improved latrines[28] available for outpatient settings or ≥ 1 per 20 users for inpatient settings?** Choose one answer	☐ Less than required number of toilets or latrines available and functioning	0
	☐ Sufficient number present but not all functioning	2.5
	☐ Sufficient number present and functioning	7.5
**Power supply, ventilation and cleaning**		
**5. In your health care facility, is sufficient energy/power supply available at day and night for all uses (for example, pumping and boiling water, sterilization and decontamination, incineration or alternative treatment technologies, electronic medical devices, general lighting of areas where health care procedures are performed to ensure safe provision of health care and lighting of toilet facilities and showers)?** Choose one answer	☐ No	0
	☐ Yes, sometimes or only in some of the mentioned areas	2.5
	☐ Yes, always and in all mentioned areas	5
**6. Is functioning environmental ventilation (natural or mechanical[29]) available in patient care areas?**	☐ No	0
	☐ Yes	5
**7. For floors and horizontal work surfaces, is there an accessible record of cleaning, signed by the cleaners each day?** Choose one answer	☐ No record of floors and surfaces being cleaned	0
	☐ Record exists, but is not completed and signed daily or is outdated	2.5
	☐ Yes, record completed and signed daily	5
**8. Are appropriate and well-maintained materials for cleaning (for example, detergent, mops, buckets, etc.) available?** Choose one answer	☐ No materials available	0
	☐ Yes, available but not well maintained	2.5
	☐ Yes, available and well-maintained	5

27 This component can be assessed in more detail using the *WHO Water and sanitation for health facility improvement tool* (WASH FIT) (http://www.who.int/water_sanitation_health/publications/water-andsanitation-for-health-facility-improvement-tool/en/, accessed13 April 2018). Particularly for these questions, the IPC team may need to consult with other relevant teams in the facility to be able to respond to questions accordingly and accurately.

28 Improved sanitation facilities include flush toilets into a managed sewer or septic tank and soak-away pit, VIP latrines, pit latrines with slab and composting toilets. To be considered usable, a toilet/latrine should have a door that is unlocked when not in use (or for which a key is available at any time) and can be locked from the inside during use. There should be no major holes or cracks or leaks in the toilet structure, the hole or pit should not be blocked, water should be available for flush/pour flush toilets. It should be within the grounds of the facility and it should be clean as noted by absence of waste, visible dirt and excreta and insects.

29 Natural ventilation: outdoor air driven by natural forces (for example, winds) through building purpose-built openings, including windows, doors, solar chimneys, wind towers and trickle ventilators. Mechanical ventilation: air driven by mechanical vans installed directly in windows or walls or in air ducts for supplying air into, or exhausting air from, a room. More information at: http://www.who.int/water_sanitation_health/publications/natural_ventilation/en/, accessed 13 April 2018.

**Patient placement and personal protective equipment (PPE) in health care settings**		
**9. Do you have single patient rooms or rooms for cohorting[30] patients with similar pathogens if the number of isolation rooms is insufficient (for example, TB, measles, cholera, Ebola, SARS)?[31]** Choose one answer	☐ No	0
	☐ No single rooms but rather rooms suitable for patient cohorting available	2.5
	☐ Yes, single rooms are available	7.5
**10. Is PPE[32] available at all times and in sufficient quantity for all uses for all health care workers?** Choose one answer	☐ No	0
	☐ Yes, but not continuously available in sufficient quantities	2.5

	☐ Yes, continuously available in sufficient quantities	7.5
**Medical waste management and sewage**		
**11. Do you have functional waste collection containers for non-infectious (general) waste, infectious waste and, sharps waste in close proximity to all waste generation points?** Choose one answer	☐ No bins or separate sharps disposal	0
	☐ Separate bins present but lids missing or more than 3/4 full; only two bins (instead of three); or bins at some but not all waste generation points	2.5
	☐ Yes	5
**12. Is a functional burial pit/fenced waste dump or municipal pick-up available for disposal of non-infectious (non-hazardous/ general waste)?** Choose one answer	☐ No pit or other disposal method used	0
	☐ Pit in facility but insufficient dimensions; pits/dumps overfilled or not fenced/locked; or irregular municipal waste pick up	2.5
	☐ Yes	5
**13. Is an incinerator or alternative treatment technology for the treatment of infectious and sharp waste (for example, an autoclave) present (either present on or off site and operated by a licensed waste management service), functional and of a sufficient capacity?** Choose one answer	☐ No, none present	0
	☐ Present, but not functional	1
	☐ Yes	5
**14. Is a wastewater treatment system (for example, septic tank followed by drainage pit) present (either on or off site) and functioning reliably?** Choose one answer	☐ No, not present	0
	☐ Yes, but not functioning reliably	2.5
	☐ Yes and functioning reliably	5
**Decontamination and sterilization**		
**15. Does your health care facility provide a dedicated decontamination area and/or sterile supply department (either present on or off site and operated by a licensed decontamination management service) for the decontamination and sterilization of medical devices and other items/equipment?** Choose one answer	☐ No, not present	0
	☐ Yes, but not functioning reliably	2.5
	☐ Yes and functioning reliably	5
**16. Do you reliably have sterile and disinfected equipment ready for use?** Choose one answer	☐ No, available on average < five days per week	0
	☐ Yes, available on average ≥ five days per week or every day, but not of sufficient quantity	2.5
	☐ Yes, available every day and of sufficient quantity	5
**17. Are disposable items available when necessary? (for example, injection safety devices, examination gloves)** Choose one answer	☐ No, not available	0
	☐ Yes, but *only* sometimes available	2.5
	☐ Yes, continuously available	5
**Subtotal score**		**/100**

30 Cohorting strategies should be based on a risk assessment conducted by the IPC team.

31 Negative pressure ventilation conditions in isolation rooms may be necessary to prevention transmission of some organisms (for example, multidrug-resistant TB).

32 Personal Protective Equipment (PPE): Medical non-sterile and surgical sterile gloves, surgical masks, goggles or face shields and gowns are considered as essential PPE. Respirators and aprons should also be available in adequate quantities in all facilities for use when necessary.

Interpretation: A three-step process

1. Add up your points

	**Score**
**Section (Core component)**	**Subtotals**
1. IPC programme	
2. IPC guidelines	
3. IPC education and training	

4. HAI surveillance	
5. Multimodal strategies	
6. Monitoring/audits of IPC practices and feedback	
7. Workload, staffing and bed occupancy	
8. Built environment, materials and equipment for IPC at the facility level	
**Final total score**	**/800**

2. Determine the assigned "IPC level" in your facility using the total score from Step 1

**Total score** (range)	**IPC level**
0–200	Inadequate
201–400	Basic
401–600	Intermediate
601–800	Advanced

Review the framework results and develop an action plan

Review the areas identified by this evaluation as requiring improvement in your facility and develop an action plan to address them. To undertake this task, consult the WHO *Interim practical manual* supporting implementation of the WHO Guidelines on Core Components of Infection Prevention and Control Programmes[2] which will provide you with guidance, templates, tips, and examples from around the world as well as with a list of relevant IPC improvement tools. Keep a copy of this assessment to compare with repeated uses in the future.
